# The effect of nigella sativa oil on healing in nasal septum perforations

**DOI:** 10.1016/j.bjorl.2025.101678

**Published:** 2025-07-29

**Authors:** Ahmet Koder, Onur Ersoy

**Affiliations:** a Trakya University Faculty of Medicine, Department of Otorhinolaryngology, Edirne, Turkey; b Trakya University Vocational School of Health Services, Department of Pathology Laboratory Techniques, Edirne, Turkey

**Keywords:** Experimental study, Nigella sativa, Nigella sativa oil, Nasal septal perforation, Wound healing

## Abstract

**Objective:**

Nasal Septal Perforation (NSP) is a challenging condition with limited treatment options, often resulting in symptoms like nasal obstruction and epistaxis. Nigella Sativa Oil (NSO), known for its anti-inflammatory and regenerative properties, has shown promise in promoting wound healing. This study aims to investigate the effects of NSO on the wound healing process in an experimental NSP model using rats, exploring its potential as a therapeutic agent for NSP treatment.

**Methods:**

Twenty-two male Sprague Dawley rats were randomly assigned to two groups. Each subject had a Nasal Septal Perforation (NSP) approximately 2 mm in diameter created. NSO (study group) and saline (control group) were applied topically once a day for 14 days. At the end of the 14th day, the rats were sacrificed, and their nasal septa were excised for macroscopic and histopathological examination. Immunohistochemically Vascular Endothelial Growth Factor (VEGF) and Proliferating Nuclear Cell Antigen (PCNA) parameters examined. Tissue morphology was evaluated, collagen density was measured. The macroscopic closure rate of the NSPs and the histopathological parameters were analyzed statistically.

**Results:**

NSO group showed significantly higher collagen density, surface morphology score, International Cartilage Repair Society (ICRS) matrix examination score, PCNA immunoreactivity and macroscopic closure rate of NSPs compared to the saline group (p < 0.05). In terms of VEGF immunoreactivity, no difference was detected between both groups (p > 0.05).

**Conclusion:**

Locally administered NSO may enhance wound healing in the nasal septum within an experimental NSP model, suggesting that NSO could be an effective alternative or adjunctive treatment for NSP, promoting better clinical outcomes.

**Level of evidence:**

Level 3.

## Highlights

• NSO significantly enhances healing in Nasal Septum Perforations (NSP) in rats.
• NSO increases collagen density and improves surface morphology, aiding structural repair.
• Higher PCNA levels in the NSO group indicate enhanced cellular proliferation.
• NSO's healing effects occur without altering VEGF levels.

## Introduction

A wound is a disruption of the normal anatomical structure and function of tissue, often caused by external trauma or injury. Wound healing encompasses processes such as varying cell proliferation, extracellular components, and the impact of soluble chemical mediators like growth factors and cytokines.^1^ It can be categorized into four major steps: coagulation, inflammatory, proliferative and reconstructive phase.^2^ During this process, various cellular and molecular mechanisms come into play to facilitate the repair and regeneration of the wounded area. Wound healing is a complex and dynamic process that aims to restore the integrity and function of damaged tissue. Understanding and optimizing wound healing is crucial for positive clinical outcomes. A suitable strategy to treat wounds is essential for positive clinical outcomes because it helps to promote healing, prevent infection, and minimize scarring.

NSP is an abnormal opening or hole in the nasal septum, the cartilage and bone that divides the nasal cavity into two nostrils. The incidence of NSP is estimated to be around 1% in the general population.^3^ NSP can occur due to various reasons such as trauma, chronic nasal infections, chronic nasal drug use, or prior nasal surgery.^4^ The most common cause of NSP is iatrogenic injuries (62.4%), especially endoscopic sinus surgery, septoplasty and septorhinoplasty.^5^ Patients with NSP often suffer from symptoms like nasal obstruction, crusting, whistling, epistaxis, and discomfort, which significantly affect quality of life.^4^^,^^6^ Current treatment options include conservative approaches (e.g., nasal lubricants, septal buttons) and surgical repair using local or regional flaps.^6^ However, recurrence rates can be high, underscoring the need for effective therapies to promote healing and prevent NSP formation. Understanding the wound healing process and identifying substances that may accelerate this process, such as NSO, could lead to improved outcomes for patients with nasal septum perforations. Nigella Sativa (NS), also known as black cumin, is a flowering plant native to the Mediterranean region and Southwest Asia.^7^ The seeds of NS contain a variety of bioactive compounds, including thymoquinone, which has been shown to possess potent antioxidant, anti-inflammatory, and wound healing properties.^7^ Studies have demonstrated the beneficial effects of NSO on various types of wounds, including cutaneous, diabetic, and surgical wounds.^7^ The active compounds in NSO have been found to enhance the proliferation of fibroblasts, increase collagen deposition, and promote angiogenesis, all of which are essential for the wound healing process.^8^ Moreover, the anti-inflammatory and antimicrobial properties of NSO may help to reduce the risk of infection and promote a favorable environment for wound healing.^9^ Given the promising evidence for the wound healing effects of NS, it is possible that NSO could also be an effective therapy for promoting the healing of nasal septum perforations.

The aim of this study was to investigate the effect of NSO on wound healing in an experimental nasal septal perforation model created in rats.

## Methods

The study was performed in accordance with the ARRIVE guidelines 2.0. This experimental study was conducted in Trakya University Experimental Animal Research Center with approvel of Trakya University's Ethics Committee for Animal Experiments (decision date/number: 25.01.2024/2024.01.02). NSO (Black Cumin Seed Oil, Zade Vital, Turkey) was obtained by cold pressing Nigella sativa seeds. The oil was stored at 4 °C in dark bottles until use.

### Animals

The minimum sample size of animals was determined by power analysis. The minimum sample size for each group, with 95% Confidence Interval, and 5% tolerable error assumptions, was 11. Twenty-two, each 200–400 g (g) weighted and aged 9–10 weaks, healthy Sprague Dawley male rats were used in this study. The animals were held in a standardized conditions with a 12:12 h light-dark light cycle and 22 ± 2 °C temperature. The animals were housed in groups of three per cage and fed ad libitum with water and standard pelleted food. The 22 animals were randomly divided into two groups: the Saline group (Control) and the NSO group (Study), with each group consisting of 11 subjects.

### Surgery

All subjects in both groups were anesthetized with Intramuscular (IM) ketamine hydrochloride (Ketalar®, Pfizer, USA) at a dose of 45 mg/kg and xylazine hydrochloride (Basilazin, Bavet, Germany) at a dose of 5 mg/kg. Ten minutes after anesthesia induction, the rats were positioned appropriately. NSP, approximately 3 mm posterior to the columella and about 2 mm in diameter, was created through the right nasal cavity using an intravenous cannula (Vasofix IV Cannula 14 G; 2.2 × 50 mm, Braun, Germany). Study group: After perforation procedure, 0.2 mL 1% NSO was applied to the right nasal cavity through a cannula. Control group: After perforation procedure, 0.2 mL saline was applied to the right nasal cavity through a cannula. NSO and saline were administered to the nasal cavity once a day at the same time by the same person for fourteen days. After each application, the animals were held in a head-up position briefly to ensure mucosal contact and prevent immediate drainage. The oil was not rinsed or removed, allowing continuous contact with the perforation site and natural absorption through the nasal mucosa. This regimen was designed to mimic a feasible clinical topical treatment protocol. At the end of the experiment, fourteen days after the surgery, the animals were sacrificed with an intraperitoneal injection of 100 mg/kg pentobarbital (Penbital, Bioveta, Czech Republic).

### Histological examination

At the end of the study, the nasal septa of the rats were excised. Each specimen was placed in separate containers without group labels. The specimens were fixed with 10% neutral buffered formalin for 24 h. Biopsy materials were embedded in paraffin after tissue follow-up procedures, and paraffin blocks were obtained. 5 μm thick sections were taken from the paraffin blocks. Masson trichrome and Sirius red stain were applied to the sections of all groups. Histopathological examinations were blindly performed by an expert histolog. Immunohistochemically, vascular endothelial growth factor (VEGF, Novus Biologicals, Littleton, CO, USA) 1/200 dilution and proliferating nuclear cell antigen (PCNA, Cell Signaling Technology, Massachusetts, USA) 1/1000 dilution primary antibodies were applied to the sections for 1 h at room temperature.

### Cell count

General tissue morphology was evaluated using Masson trichrome stained slides. Collagen density was measured with the Imaging Analysis System (Version 2.11.5.1, Kameram-Argenit, Istanbul, Turkey) using Sirius red stained slides. The density of collagen fibres over the total area is given as %.^10^

Tissue morphology was determined according to the ICRS histological evaluation scale Table 1, Table 2.^11^ The macroscopic closure of the septal perforations was scored in five categories (0: Increased too much, 1: Slightly increased, 2: Unchanged, 3: Partially closed, 4: Completely closed).

**Table 1:** ICRS histological surface score (n = 11).

Surface morphology	Score
Smooth, continuous	3
Discontinuous, irregular	0

**Table 2:** ICRS histological matrix score (n = 11).

Matrix feature	Score
Hyaline	3
Hyaline/Fibrocartilage	2
Fibrocartilage	1
Fibrous tissue	0

The number of PCNA positive cells was determined as a ratio (%) to the total number of cells per 0.02 mm^2^ area.^12^

VEGF immunoreactivity was evaluated semiquantitatively using the HSCORE method.^13^

### Statistical analysis

The minimum sample size was determined using the G* Power program. Statistical analyzes were performed using SPSS 20.0 program (License number: 10240642) at Trakya University Faculty of Medicine, Department of Biostatistics and Medical Informatics, and values were taken as mean ± Standard Deviation (SD); p < 0.05 value was considered significant. Pairwise comparisons for differences between groups were determined by the Mann-Whitney *U* test.

## Results

**Fig. 1:**
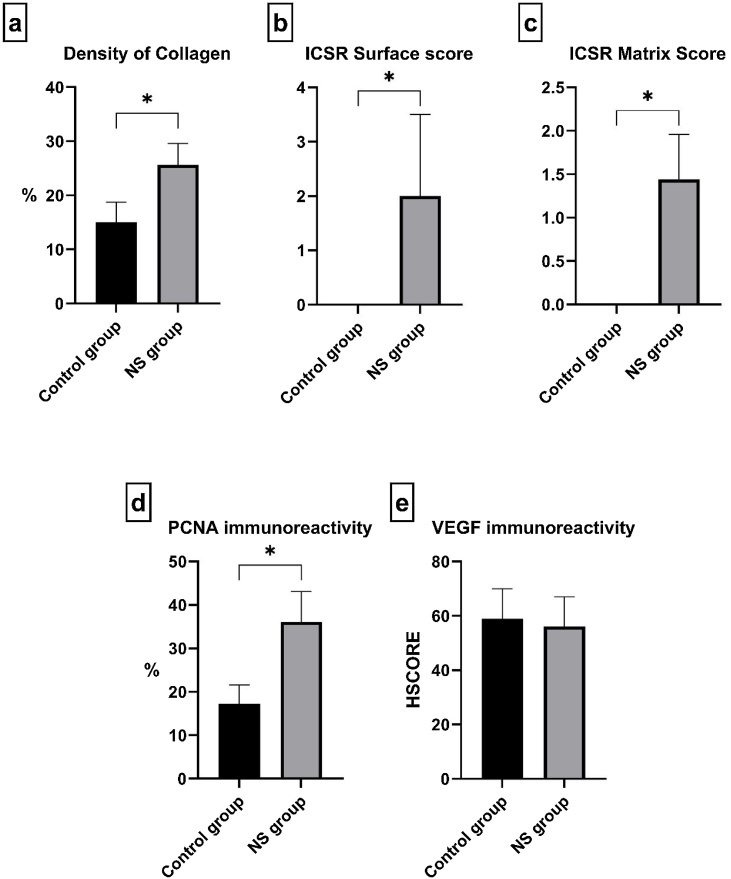
Graphic (a) density of collagen, Graphic (b) ICSR surface score, Graphic (c) ICSR matrix score, Graphic (d) PCNA immunoreactivity, Graphic (e) VEGF immunoreactivity. p < 0.05 value was considered significant. Pairwise comparisons for differences between groups were determined by the Mann-Whitney *U* test.

**Fig. 2:**
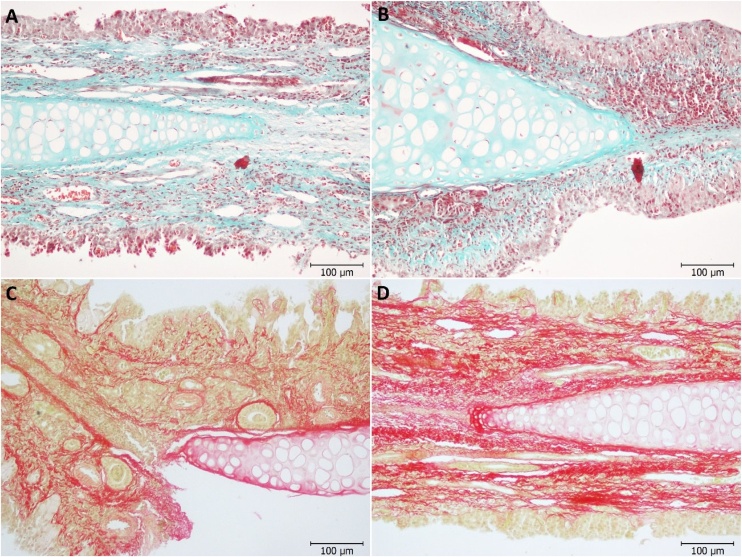
Masson trichrome staining (A) Control group, (B) NS group, Sirius red staining (C), Control group, (D) NS group.

A significantly greater amount of newly formed tissue collagen was observed in the NSP group compared to the control group (p < 0.001, Fig. 1a). Collagen density is observed between groups in Sirius red and Masson stained preparations (Fig. 2A‒D). When the amount of newly formed tissue collagen in the perforation area was evaluated, a greater amount of collagen development was observed in the NSO group compared to the control group (p < 0.001, Fig. 1a). Collagen density is observed between groups in Sirius red and Masson stained preparations (Fig. 2A‒D).

According to the ICRS histological surface morphology examination results, a discontinuous and irregular surface was detected in all samples in the control group. In the NSO group, the surface was found to be smooth and uninterrupted, except for 3 samples. When the two groups were compared statistically, the surface morphology score was found to be higher in the NSO group than in the control group (p = 0.014, Fig. 1b). ICRS matrix examination score was found to be higher in the NSO group than in the control group (p < 0.001, Fig. 1c). The excess fibrocartilage tissue in the NSO group is striking.

**Fig. 3:**
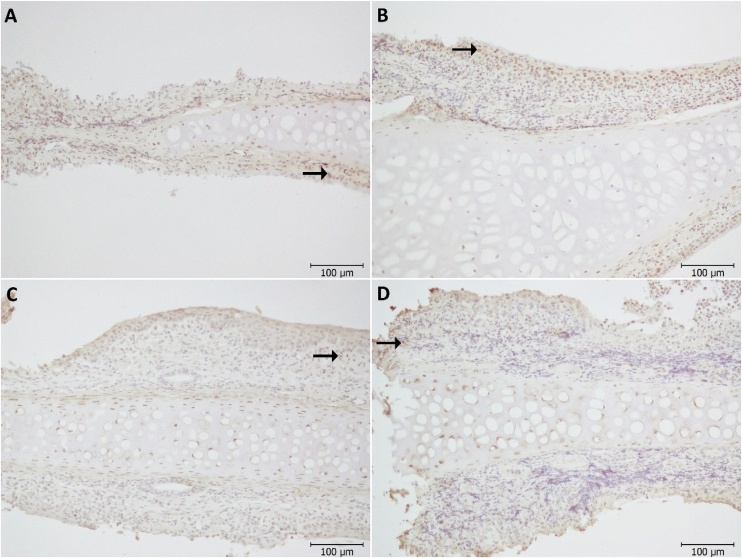
PCNA immunoreactivity (A) Control group, (B) NS group, VEGF immunoreactivity (C) Control group, (D) NS group. Positive cells was shown (→).

PCNA immunoreactivity was detected significantly higher in the NSO group than in the control group (p < 0.001, Fig. 1d). PCNA immunoreactivity is observed in more nuclei in the epithelial tissue, especially in the NSO group, compared to the control group (Fig. 3A‒B). In terms of VEGF immunoreactivity, no difference was detected between both groups (p = 0.605, Fig. 1e). VEGF immunoreactivity observed cytoplasmically is observed between the groups (Fig. 3C‒D).

**Fig. 4:**
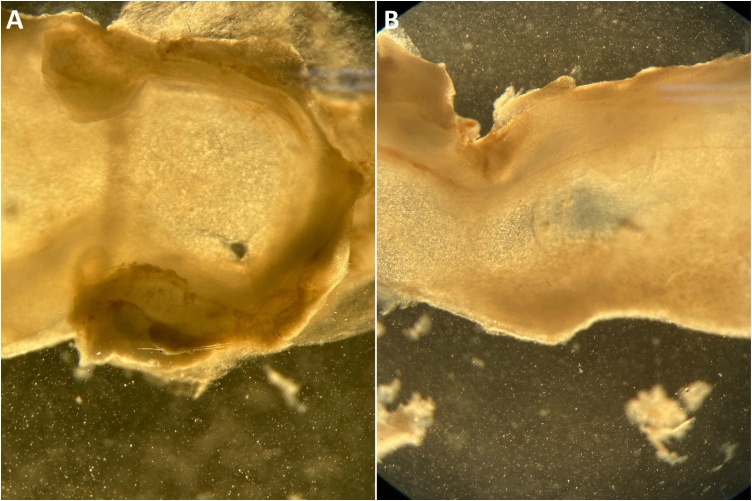
Macroscopic image of perforation area. (A) Control group, (B) NS group.

Nasal septal perforations were fully closed in all rats within the NSO group. In contrast, within the control group, 5 animals (45.4%) had complete closure of the perforations, 3 animals (27.2%) experienced partial closure, 3 animals (27.2%) showed no change in perforation size, the macroscopic closure rate of the nasal septal perforations was significantly higher in the NSO group (p = 0.015, p < 0.05). Macroscopic image of perforation area (Fig. 4).

## Discussion

Nasal septal perforations can have a variety of underlying causes, with the most common being previous nasal surgeries, especially septoplasty, septorhinoplasty, and endoscopic sinus surgery. Trauma, chronic nasal infections, chronic nasal irritation, and intranasal drug use can also contribute to the development of nasal septal perforations.^3^ Symptoms of nasal septal perforations can include nasal crusting, epistaxis, nasal whistling, nasal obstruction, and a sense of nasal obstruction.^14^ Larger perforations can lead to more significant issues, such as nasal deformity, nasal collapse, and difficulty with nasal airflow.^15^ The negative effects of nasal septal perforations on human health and quality of life can be significant. Patients may experience recurrent nasal bleeding, chronic nasal crusting, and a whistling or bubbly sound during breathing.^15^ These symptoms can lead to social embarrassment, difficulty with work or school, and decreased quality of life. The treatment of nasal septal perforations can be challenging. Medical treatments, such as the use of nasal ointments, lubricants, or humidifiers, can help to alleviate symptoms, but they do not address the underlying perforation. Surgical treatment, such as perforation closure or reconstruction, is often necessary for more significant perforations. However, the success rates of these surgical interventions can be variable, and they are associated with a risk of complications, such as persistent perforation, nasal obstruction, and nasal deformity.^16^ One of the main challenges of treating nasal septal perforations is the risk of recurrence. Even after successful surgical repair, the perforation can reopen due to factors such as poor wound healing, persistent nasal irritation, or underlying medical conditions. Additionally, the surgical repair of nasal septal perforations can be technically challenging, with a high risk of complications, such as persistent nasal obstruction, septal deformity, and anosmia. To prevent the development of nasal septal perforations during surgeries, such as septoplasty, septorhinoplasty, and endoscopic sinus surgery, it is crucial for surgeons to minimize tissue trauma and ensure proper wound healing. In this experimental study, we hypothesized that NSO improves wound healing in the nasal septum. We developed a NSP model on rats to test this hypothesis. Our results demonstrated that NSO positively affects wound healing by enhancing collagen density, more nuclei in the epithelial surface and surface morphology examination found smooth and uninterrupted at the study group.

NS, also known as black cumin, is a plant with a long history of medicinal use. NSO has shown promising benefits for wound healing due to its potent antioxidant, anti-inflammatory, and tissue regenerative properties.^7^ NS is classified as “generally recognized as safe” by the FDA in the United States and is available in various therapeutic formulations, including dietary supplements, oils, topical creams, and powders.^17^ NSO contains a diverse array of bioactive compounds, including thymoquinone, nigellone, and p-cymene, which have been extensively studied for their ability to promote various stages of wound healing.^7^^,^^18^ Moreover, the topical application of NSO has been found to be safe and well-tolerated, with minimal adverse effects reported in clinical studies.^19^ The pathophysiology of wound healing involves a complex cascade of cellular and molecular processes, including hemostasis, inflammation, proliferation, and remodeling.^7^ NSO has been demonstrated to accelerate these stages by enhancing angiogenesis, collagen deposition, and modulating the inflammatory response.^18^, ^19^, ^20^

The inflammatory phase, whose primary purpose is to prevent wound infection, begins immediately after an injury.^21^ During this phase, leukocytes, particularly neutrophils, migrate to the wound site and start producing Reactive Oxygen Species (ROS).^21^^,^^22^ The increase in ROS can disrupt cellular functions, leading to cell death and potentially impairing wound healing. NSO may improves wound healing at this phase with its anti-infammatory antibacterial antifungal properties.^23^ A study concludes that NSO enhances wound healing through its anti-inflammatory properties.^24^ NSO contains active compounds like thymoquinone, dithymoquinone, thymohydroquinone, and thymol, all of which exhibit strong antibacterial effects.^25^ In fact, it has demonstrated wound healing potential by regulating inflammatory markers (NF-B, TNF-α, and ILs) and upregulating growth factor expression (VEGF and TGF-β), thereby modulating collagen-1 expression to promote angiogenesis.^26^^,^^27^ One proposed mechanism behind the bactericidal ability of NSO is its weakening effect on the integrity of bacterial membranes.^28^

During the proliferative phase, which is a crucial step in wound healing, epithelial cells migrate from the wound edges, proliferate, and cover the wound.^21^ This process, marking the beginning of epithelialization, is regulated by various factors, including Transforming Growth Factor (TGF), Epidermal Growth Factor (EGF), and Fibroblast Growth Factor (FGF).^29^, ^30^, ^31^ The therapeutic use of NSO as a wound-healing agent has resulted in improved fibroblast formation, increased granulation tissue production, enhanced wound contraction, and re-epithelialization.^8^^,^^32^^,^^33^ One of the initial and most critical steps of the proliferative phase is angiogenesis, which is induced by factors such as TGF-α, TGF-β, FGF, PDGF, and Vascular Endothelial Growth Factor (VEGF).Studies have shown that NSO can significantly increase the expression of growth factors, such as Vascular Endothelial Growth Factor (VEGF) and Transforming Growth Factor-beta (TGF-β), which are crucial for tissue regeneration and wound healing.^8^ In our study, however, no difference in VEGF levels was found between the two groups. Although VEGF immunoreactivity did not differ significantly between groups in our study, NSO appears to promote wound healing through alternative mechanisms. Thymoquinone, the primary active compound in NSO, exerts potent anti-inflammatory and antioxidant effects, reduces neutrophil infiltration, and enhances fibroblast proliferation and collagen synthesis. These effects contribute to tissue regeneration independently of VEGF. In vitro and in vivo studies have shown that NSO can upregulate other growth factors such as TGF-β, PDGF, and bFGF, which are also essential in the wound healing cascade. Therefore, the absence of VEGF upregulation does not preclude the regenerative potential of NSO in nasal septal wounds.^7^^,^^8^^,^^34^

Collagen synthesis plays a crucial role in all phases of wound healing.^35^ Collagen produced by fibroblasts is a key component of the Extracellular Matrix (ECM). The rate of ECM synthesis, along with the quantity and quality of the produced matrix, directly influences scar formation.^20^^,^^36^^,^^37^ Previous studies have shown that NSO increases the collagen synthesis.^7^ In our study, a greater amount of collagen development was also observed in the NSO group compared to the control group (p < 0.001). The ICRS histological examination revealed a discontinuous and irregular surface in all control group samples. In the NSO group, the surface was smooth and uninterrupted in all except 3 samples. Statistically, the NSO group had a higher surface morphology score than the control group (p < 0.001). The ICRS matrix score was also higher in the NSO group. PCNA is an ancient molecule that plays a crucial role in sustaining life by facilitating DNA replication and repair.^38^ It is well-known nuclear protein, clinically trusted marker for proliferation, which participates in cell proliferation by mediating DNA polymerase.^39^ In our study, PCNA immunoreactivity was significantly higher in the NSO group compared to the control group.

NSO, rich in thymoquinone, promotes wound healing through anti-inflammatory, antioxidant, and regenerative effects. It reduces pro-inflammatory cytokines (e.g., TNF-α, IL-1β) and enhances antioxidant enzymes like SOD and catalase, minimizing tissue damage.^7^ NSO also stimulates fibroblast proliferation, collagen synthesis, and re-epithelialization.^8^ Additionally, NSO has been shown to upregulate growth factors such as VEGF and PDGF, contributing to angiogenesis and tissue remodeling.^34^ These combined actions support NSO’s therapeutic potential in accelerating wound repair. Management of nasal septal perforation typically includes conservative approaches such as nasal irrigation, emollients, or silicone septal buttons, which aim to relieve symptoms but do not promote tissue regeneration. Surgical repair remains the definitive treatment, though it is technically challenging, invasive, and associated with recurrence in large defects. In contrast, Nigella sativa oil offers a minimally invasive alternative with biological activity. Its anti-inflammatory and tissue-regenerative properties, including enhanced collagen synthesis and mucosal healing, suggest potential utility in promoting perforation closure, particularly in small-to-moderate defects.

In this study, we demonstrated the positive effects of NSO during the proliferative and remodeling phases, showing significantly higher values in PCNA immunoreactivity, surface morphology, collagen density, and macroscopic closure rate (p < 0.05). This experimental study, which is the first in the literature to demonstrate the positive effects of NSO on nasal septal wound healing, has several limitations. Firstly, using a cream form instead of an oil form might have allowed it to stay on the wound site longer. The second limitation is the small group sizes, which were determined based on the ethical “principle of reduction”. However, the sample size remains statistically sufficient. Additionally, the short duration of the study may not have fully captured the long-term effects of NSO on wound healing.

Future studies should aim to include larger sample sizes and longer treatment durations to better assess the long-term regenerative potential of NSO. Dose-response investigations may also help define the most effective application parameters. Ultimately, well-designed clinical trials are needed to evaluate the feasibility and therapeutic value of NSO in human patients with nasal septal perforations.

## Conclusion

This study demonstrates the significant therapeutic potential of NSO in enhancing the healing of NSP. The findings suggest that NSO could be an effective alternative or adjunctive treatment for NSP, promoting better clinical outcomes. Given these promising results, NSO could be considered a potential therapeutic agent for managing NSP in clinical settings. However, further studies are needed to elucidate the precise molecular mechanisms and to evaluate the long-term effects and safety of NSO in humans.

## Funding

No funding.

## Declaration of competing interest

The authors declare no conflicts of interest.
